# Matrix metalloprotease-9 modulates microglial/macrophage responses in murine brain demyelination

**DOI:** 10.1186/s40478-026-02371-2

**Published:** 2026-07-10

**Authors:** Eline Bernaerts, Amber De Visscher, Sam Vanherle, Marie Rutten, Fran Prenen, Nele Berghmans, Jana Van Broeckhoven, Hanne Meers, Mirre De Bondt, Febe Boudry, Sofie Knoops, Tania Mitera, Lien De Somer, Jeroen Bogie, Patrick Matthys, Jennifer Vandooren

**Affiliations:** 1 Laboratory of Immunobiology, Rega Institute for Medical Research, KU Leuven, 3000 Leuven, Belgium; 2 Department of Immunology and Infection, Biomedical Research Institute, Hasselt University, 3590 Diepenbeek, Belgium; 3 University MS Center, 3900 Pelt, Belgium; 4 Laboratory of Immunoparasitology, Rega Institute for Medical Research, KU Leuven, 3000 Leuven, Belgium; 5 Laboratory of Molecular Immunology, Rega Institute for Medical Research, KU Leuven, 3000 Leuven, Belgium; 6 Department of Microbiology, Immunology and Transplantation, KU Leuven Campus Kulak, 8500 Kortrijk, Belgium; 7 Laboratory of Pediatric Immunology, University Hospital Leuven, 3000 Leuven, Belgium

**Keywords:** Microglia, Lysophosphatidylcholine model, Cuprizone-induced demyelination model, Proteolysis, MMP-9, Inflammation, Resolution

## Abstract

Multiple sclerosis (MS) is a chronic autoimmune disorder, in which the immune system targets the protective myelin sheath surrounding axons in the brain, spinal cord and optic nerve, leading to demyelination and ultimately neurodegeneration. Promoting remyelination to delay or halt disease progression remains a major therapeutic challenge in MS research. Matrix metalloproteases (MMPs) have been implicated in the pathogenesis of MS. Elevated levels of MMP-9 have been detected in cerebrospinal fluid (CSF), serum and demyelinating lesions of MS patients, where MMP-9 contributes to myelin breakdown, epitope generation, and leukocyte infiltration. In the in vivo experimental autoimmune encephalomyelitis and lysophosphatidylcholine (LPC) murine MS models, *Mmp9*^*−/−*^ mice exhibited delayed resolution of disease symptoms, suggesting a role for MMP-9 in remyelination. In this study, we investigated the role of MMP-9 in demyelination and remyelination, using two complementary models: LPC-induced demyelination in ex vivo brain slices and cuprizone (CPZ)-induced demyelination in vivo. In LPC-treated slices, MMP-9 deficiency impaired remyelination via microglia/macrophage-mediated mechanisms. Consistently, *Mmp9*^*−/−*^ mice displayed increased numbers of activated microglia/macrophages in the corpus callosum following CPZ intoxication, with modest persistence during remyelination compared to *WT* mice. Naïve *Mmp9*^*−/−*^ microglia/macrophages also showed enhanced myelin debris phagocytosis compared to *WT* cells. However, MMP-9 deficiency had minimal impact on demyelination or remyelination in the CPZ model. Proteomic analysis of CSF revealed differential expression of inflammatory mediators, including decreased levels of CCL2 and CXCL9, whereas CCL20 was increased in *Mmp9*^*−/−*^ CPZ-treated mice. Collectively, these findings indicate that MMP-9 does not directly regulate demyelination and remyelination in the CPZ-induced demyelination model, but increases microglial abundance, highlighting its indirect role in CNS demyelination.

**Supplementary Information:**

The online version contains supplementary material available at https://doi.org/10.1186/s40478-026-02371-2.

## Introduction

Multiple sclerosis (MS) is a chronic neuroinflammatory autoimmune disease, characterized by immune-mediated damage to the myelin sheath surrounding axons in the brain, spinal cord, and optic nerve. This demyelination disrupts the conduction of electrical impulses along nerve fibers, leading to neurological deficits and progressive disability [1–4]. In the central nervous system (CNS), myelin is produced by mature oligodendrocytes and tightly wrapped around axons. Its structural integrity relies on key proteins such as myelin basic protein (MBP) and proteolipid protein (PLP). However, inflammatory conditions can lead to oligodendrocyte loss, resulting in myelin degradation and axonal injury. In response to demyelination, the CNS initiates remyelination—a regenerative process in which oligodendrocyte precursor cells (OPCs) differentiate into mature oligodendrocytes capable of synthesizing new myelin. Although remyelination is often efficient during the early stages of MS, it becomes incomplete or fails entirely in chronic lesions. Therefore, promoting remyelination remains a major therapeutic challenge in MS research [5, 6].

Matrix metalloprotease (MMP)-9 has emerged as a key player in MS pathogenesis, supported by clinical and preclinical evidence. In MS patients, elevated MMP-9 levels have been detected in brain lesions [7–10], cerebrospinal fluid (CSF) [11, 12] and peripheral blood mononuclear cells (PBMCs) [12–15], which correlate with disease activity and lesion formation [16–18]. In the experimental autoimmune encephalomyelitis (EAE) model, MMP-2 and MMP-9 activity has been observed at sites of parenchymal border penetration via in situ zymography [19]. One of the earliest pathological events in MS is blood-brain barrier (BBB) disruption, which facilitates the infiltration of blood-derived leukocytes into the CNS parenchyma, contributing to focal lesion development [20]. Preclinical studies have shown that MMPs compromise BBB integrity by degrading tight junction [21] and basal lamina proteins, including dystroglycan [22] and N-cadherin [23]. Additionally, MMPs enhance leukocyte migration from perivascular cuffs into the CNS parenchyma by modulating chemotactic signals [19]. Mice treated with protease inhibitors [7, 24–27] and young *Mmp9*^*−/−*^ mice [38] are less susceptible to EAE induction and showed milder clinical symptoms, further highlighting a central role for MMP-9 in disease initiation. A clinical trial also demonstrated that minocycline, a tetracycline antibiotic with broad-spectrum MMP-inhibitory properties, reduces gadolinium-enhancing lesions in patients experiencing their first MS attack, thereby limiting leukocyte infiltration into the CNS [28, 29].

Beyond its role in BBB disruption, MMP-9-mediated proteolysis has been linked to myelin fragmentation, primarily due to increased MMP-9 levels in MS brains with enhanced myelin sheath degradation and the release of immunogenic peptides [30–34]. In addition, in vitro studies showed that MMPs are able to directly degrade MBP [33]. However, *Mmp9*^*−/−*^ mice subjected to lysophosphatidylcholine (LPC) model, which induces focal demyelination, did not show differences in demyelination compared to *WT* controls, but exhibited impaired remyelination through cleavage of inhibitory neural glial antigen 2 [35]. Similarly, *Mmp9*^*−/−*^ EAE mice displayed an attenuated disease onset and a prolonged resolution compared to *WT* mice, suggesting a dual role for MMP-9 in both the initiation and resolution phases of neuroinflammation [36].

A deeper understanding of the role of MMP-9 in demyelination and remyelination is essential for developing strategies to remodel the MS lesion environment and enhance remyelination. To this end, we investigated the impact of MMP-9 genetic deletion on demyelination and remyelination, using two complementary models: LPC-induced demyelination in ex vivo brain slices and cuprizone (CPZ)-induced demyelination in vivo, both occurring independently of peripheral immune cell infiltration [37].

## Materials and methods

### Cerebellar brain slice de-/remyelination model

Cerebellar brain slices were obtained from *WT* and *Mmp9*^*−/−*^ C57BL/6J mouse pups at postnatal day 10 (P10). Brain slices were cultured in MEM medium (Cat # 32360-026,Thermo Fisher Scientific) supplemented with 25% horse serum (Cat # 10368-902, Thermo Fisher Scientific), 25% Hank’s balanced salt solution (Cat # H6648, Sigma-Aldrich), 50 U/mL penicillin, 50 U/mL streptomycin, 1% Glutamax (Cat # 35050-038, Thermo Fisher Scientific), 12.5 mM HEPES (Cat # 15630-080, Thermo Fisher Scientific) and 1.45 g/L glucose (Cat # G8644, Sigma-Aldrich). Phagocytes were depleted by treating slices with clodronate or empty liposomes (0.5 mg/mL, Cat # CP-005-005, LIPOSOMA) immediately after isolation for 24 h. To induce demyelination, brain slices were treated with 0.5 mg/mL LPC (Cat # L4129, Sigma-Aldrich) at 3 days post isolation for 16 h. Next, brain slices were allowed to recover in culture medium for 1 week to study remyelination, followed by histological and biochemical analysis.

### Cuprizone-induced demyelination model

*WT* and *Mmp9*^*−/−*^ C57BL/6J mice were developed and bred at the Rega Institute for Medical Research [38] (Leuven, Belgium).

The CPZ-induced demyelination model, further referred to as the CPZ model, was induced by feeding *WT* and *Mmp9*^*−/−*^ mice, weighing between 18 and 22 g at the age range of 7–10 weeks, *ad libitum* a diet containing 0.3% (w/w) cuprizone (bis[cyclohexanone] oxaldihydrazone, Cat # 1544210002, Acros Organics) mixed in powdered standard rodent chow, further referred to as CPZ. A concentration of 0.3% (w/w) is commonly used to induce robust and consistent demyelination in C57BL/6 mice. Mice were monitored regularly for body weight and general health, and no excessive morbidity or mortality was observed under the 0.3% (w/w) CPZ regimen. To investigate demyelination, mice were sacrificed at day 28 (severe demyelination). To investigate remyelination, mice were fed with standard pellet rodent chow for 1 week after CPZ withdrawal to induce spontaneous remyelination and were sacrificed at day 42. Weight-matched control mice were fed with ground standard rodent chow without CPZ, further referred to as ND. Experiments were approved by the Ethics Committee of KU Leuven (P046/2021).

### Visual evoked potential

Epidermal visual evoked potential (VEP) measurements were conducted at different timepoints: baseline (before CPZ diet), 4 weeks after CPZ diet, and following 5 weeks of CPZ and 1 week of normal chow. Animals were dark-adapted for at least 2 h prior to recordings. The procedure was performed using the Celeris system and ESPION software. Mice were anesthetized by intraperitoneal (i.p.) injection of ketamine (80 mg/kg) and xylazine (20 mg/kg). Pupils were dilated using 1% tropicamide for 5 min and 2.5% phenylephrine hydrochloride for 1 min. Electrodes were placed subdermally at the visual cortex, base of the tail for grounding, tongue for reference, and cornea for flash stimulus. Vidisic eye ointment was applied to prevent dryness. After verification of electrode impedance, mice were stimulated with 200 white light flashes (310 ms duration, 1 Hz frequency, 0.5 cds/m² intensity). Latency time, defined as the interval between the visual stimulus and signal detection in the visual cortex, was measured in milliseconds as a readout of visual pathway myelination.

### Immunofluorescence

Mice were euthanized by a single i.p. injection of 100 µL dolethal (Vétoquinol, 200 mg/mL) and whole brains were dissected. Whole brains were fixed in 4% paraformaldehyde (PFA, Cat # 047377.9 M, ThermoFisher Scientific) for 5 h. Fixed brains were freeze-protected in 30% sucrose (Cat # 220900010, Acros) solution for 48 h. Dehydrated brains were snap frozen in 2-methylbutane (Cat # 126470010, ThermoFisher Scientific) and stored at – 80 °C until further processing. Brains were sectioned in 16 μm thick cryosections at the level of the stereotaxic coordinates Bregma − 1.22 until Bregma − 1.94 provided by Franklin and Paxinos, using a cryostat. Slices were incubated with permeabilization buffer [0.1% Triton X-100 (Cat # 9002-93-1, ThermoFisher Scientific) in HBSS buffer [10X HBSS Ca/Mg (Cat # 14065-049, ThermoFisher Scientific) supplemented with 1 g/L bovine serum albumin (BSA, Cat # T8844.4, Carl Roth) and 0.35 g/L NaHCO_3_ (Cat #13433, Honeywell Riedel-de-Haën)] for 1 h. Thereafter, slices were blocked in HBSS buffer containing 10% fetal bovine serum (FBS, Cat # DE14-80FF, Lonza) and 1% murine FcR Blocking Reagent (Cat # 130-092-575, Miltenyi Biotec) for 2 h. Immunofluorescent analysis was performed using a rabbit anti-IBA1 antibody (1:500, Cat # 019-19741, WAKO), rat-anti MBP (1:750, Cat # MAB386, EMD Millipore) in combination with secondary RXX-conjugated donkey anti-rabbit (Cat # 711-295-152, Jackson ImmunoResearch), secondary donkey anti-rat AF488 (Cat #712-545-150, Jackson ImmunoResearch). Cell nuclei were visualised using Hoechst 33342 (10 µg/mL, Cat #62249, ThermoFisher Scientific). To preserve fluorescence, brain sections were mounted with ProLong Diamond Antifade Mountant (Cat # P36961, Invitrogen). Unspecific secondary antibody bindings to the tissue itself were excluded by incubating sections with the fluorescent secondary antibodies alone. Fluorescence was detected by confocal microscopy (Leica DMi8, Dragonfly 200, Andor). Quantification was performed with ImageJ and Imaris software. For each mouse, the presence of IBA1 + cells in the midbrain corpus callosum (CC) was determined in 4 brain slices.

To stain cerebellar brain slices, samples were incubated with relevant primary antibodies diluted in blocking buffer (1x PBS + 5% horse serum + 0.3% Triton X-100). The following antibodies were used: anti-CC1 (1/50, Cat # ab16794, Abcam), anti-olig2 (1/50, Cat # AF2418, R&D systems), anti-NF (1/1000, Cat # Ab8135, Abcam), anti-MBP (1/250, Cat # MCA409S, Millipore), anti-F4/80 (1/100, Cat # MCA497G, Bio-Rad). BODIPY (2 µM, Cat # D3922, Thermo Fisher Scientific) was used to stain neutral lipids. Analysis of cerebellar brain was done with the LSM 900 confocal microscope (Zeiss). ImageJ was used to define OLIG2 + and CC1 + cells in the cerebellar brain slices, which is presented as the percentage OLIG2 + CC1+ cells/CC1 + cells, and the myelination index (MBP + NF+ axons/NF+ axons) which is presented in a relative normalized manner and the lipid load in phagocytes, which is presented as a percentage of BODIPY+ area in F4/80 + cells.

### Oil red O staining

To visualize myelin, unfixed cryosections were stained with 0.3% Oil Red O (ORO) (Sigma-Aldrich) for 10 min. Cryosections were counterstained with hematoxylin (Merck). Images were acquired using the EVOS M3000 Imaging system (ThermoFisher Scientific). ImageJ was used to align the CC and to determine the ORO+ area in this alignment.

### Isolation of brain cells

Mice were euthanized by a single i.p. injection of 100 µL dolethal (Vétoquinol, 200 mg/mL). Mice were transcardially perfused with 25 mL PBS and whole brains were dissected. CC and cortex were surgically dissected between the stereotaxic coordinates Bregma − 0.94 and Bregma − 2.30 (see Suppl. Fig. S1). To generate single cell suspensions, we used the Adult Brain Dissociation kit mouse and rat (Cat # 130-107-677, Miltenyi Biotec) according to the manufacturer’s protocols. Briefly, CC and cortex were incubated with the required enzymes mixtures for a total of 45 min at 37 °C to obtain complete dissociation. Myelin debris was removed using the Debris Removal Solution and red blood cells were lysed using 1X Red Blood cell Removal Solution. Upon cell isolation, cells were counted and resuspended in appropriate buffer for further applications.

### Flow cytometry

Brain cells were preincubated with murine FcR blocking reagent (1:100, Cat # 130-092-575, Miltenyi Biotec) and anti-FcγRIV antibody (1:100, clone 9E9, Cat #149502, Biolegend) to block nonspecific binding of antibodies. To distinguish living cells from dead cells, samples were stained with Zombie Aqua516 (1:1000, Cat # 423101, Biolegend). For staining of extracellular markers, cells were incubated in the dark at 4 °C during 25 min with an antibody master mix of fluorochrome-labeled antibodies in FACS buffer [PBS with 2% FCS and 2mM ethylenediaminetetraacetic acid (EDTA)] supplemented with brilliant stain buffer (Cat # 563794, BD Biosciences), and with additional rat serum to block nonspecific binding of antibodies. A list of the used antibodies can be found in Suppl. Table S1. Flow cytometric analysis was performed on a BD LSR Fortessa X20 with DIVA software and data were analyzed using FlowJo v10.9 (FlowJo, LLC). Absolute cell counts of each cell type were determined by multiplying the frequency of this cell type among the living cells with the total cell count determined with the Bürker chamber.

### CSF collection and Olink^®^ platform

Mice were sedated by an i.p. injection of 50 µL xylazine-ketamine solution [xylazine: ketamine: PBS 1:3:4, (Cat # BE-V170581, VDM Livestock pharma), (Cat # BE-V439546, Dechra Pharmaceuticals)]. CSF was collected at the cisterna magna and centrifuged at 350 x g for 10 min at 4 °C to obtain cell free-CSF. All CF-CSF samples were randomized and plated at the Rega institute for Medical Research before delivery to the Olink platform at UMC Utrecht. 5 µL of each sample were run on the Olink^®^ target 96 mouse exploratory panel in a next-generation sequencing automated workflow to test 96 assays with a specificity of 99.5%. Relative quantification of each protein was represented in terms of log_2_ normalized protein expression (NPX) values.

### Quantitative RT-PCR

#### Whole brain

Quantitative RT-PCR (qRT-PCR) was performed on whole brains and isolated brain cells. Whole brains were mechanically lysed in RIPA buffer supplemented with β-mercaptoethanol using the Precellys tissue homogenizer, after which RNA was purified using the PureLink RNA Mini kit (Cat # 12183018A and 12183025, Invitrogen) according to the manufacturer’s instructions. Brain cells were lysed and RNA was isolated using RNeasy mini kit (Cat # NC9677589, Qiagen) according to the manufacturer’s instructions. For each sample, cDNA was synthesized using the High capacity cDNA reverse transcription kit (Cat # 4368814, ThermoFisher Scientific) and qRT-PCR was performed in duplicates with primer and probe sets from Integrated DNA Technologies (IDT) using an ABI Prism 7500 sequence detection system (Applied Biosystems). Data was normalized to the 18 S RNA levels as a housekeeping gene and subsequently expressed relative to the ND control group. Primers used for qRT-PCR are summarized in Suppl. Table 2.

### Phagocytosis of myelin debris

#### Microglia purification

Microglia/macrophages were enriched from isolated mouse brain cells using the EasySep Mouse CD11b positive Selection kit II (Cat # 18970, STEMCELL Technologies), according to the manufacturer’s protocols. CD11b positive cells were considered to be microglia/macrophages and were plated in a 96-well plate (50,000 microglia/well) and incubated with Calcein AM (1 µM, Cat # C1430, Invitrogen) to stain for cell viability.

#### IncuCyte analysis

Purified myelin from post-mortem healthy mouse brain was generously provided by the laboratory of Prof. Jeroen Bogie (UHasselt). Myelin was labelled with pHrodo SE (0.02 µL/µg myelin; Cat # 36600, Invitrogen) for 30 min at RT. To remove excess of pHrodo, the sample was washed twice with PBS. pHrodo-labelled myelin (100 µg/mL) was added to microglia/macrophages. Plates were imaged every 30 min in the IncuCyte S3 system (Sartorius) for 6 h and analyzed with IncuCyte analysis software (Sartorius). The number of phagocytic events (i.e. the number of Calcein AM and pHrodo-red fluorescent overlapping events) were determined for each timepoint in every well.

#### SDS PAGE and Western blot

For Western blot analyses, CC were mechanically lysed in RIPA buffer supplemented with 1% phosphatase inhibitor 2 (Cat # P5726, Sigma-Aldrich), 1% phosphatase inhibitor 3 (Cat # P0044 Sigma-Aldrich), 1 mM PMSF (Cat # 36978, ThermoFisher Scientific) and 1% HALT protease inhibitor (Cat # 78437, ThermoFisher Scientific) using the Precellys tissue homogenizer. Protein concentrations were determined with BCA protein kit (Cat # A55864, ThermoFisher Scientific). Equal amounts of protein were resuspended in reducing loading buffer (125 mM Tris-HCl pH 6.8, 10% β-mercaptoethanol, 4% SDS, 20% glycerol, 0.1% bromo-phenol blue) and incubated for 10 min at 80 °C. Samples were separated in 4–20% Tris-Glycine gels. Proteins in gels were transferred onto PVDF membranes using the Trans-Blot Turbo Transfer system with associated materials and protocols (Cat # 1704150, Biorad). After protein transfer, membranes were blocked for 5 min in EveryBlot Blocking buffer (Cat # 12010020, Biorad) and incubated with anti-MBP (Cat # MCA409S, Biorad), anti-β-tubulin (Cat # MA5-16308-HRP, Proteintech) antibodies diluted in non-fat dry milk (NFDM) or 10% EveryBlot Blocking buffer in TBS-T (1× TBS containing 0.1% Tween 20) overnight at 4 °C. Membranes were washed three times with TBS-T at RT and incubated for 1 h with secondary anti-rat IgG HRP-conjugated antibody solution IgG (Cat # PI-9400, Vector Labs) at RT. Membranes were washed three times with TBS-T and bands were visualized using the Pierce ECL substrate (Cat # 32106, ThermoFisher Scientific). Western blots were imaged using the Vilber Lourmat Fusion system (Labtech International).

#### Sandwich ELISA

For ELISA analyses, CC were mechanically lysed in N-PER tissue protein extraction reagent (Cat # 87792, ThermoFisher Scientific) supplemented with 1% phosphatase inhibitor 2 (Cat # P5726, Sigma-Aldrich), 1% phosphatase inhibitor 3 (Cat # P0044 Sigma-Aldrich), 1 mM PMSF (Cat # 36978, ThermoFisher Scientific) and 1% HALT protease inhibitor (Cat # 78437, ThermoFisher Scientific), using the Precellys tissue homogenizer. Protein concentrations were determined with BCA protein. APP (Mouse amyloid-beta precursor (APP) ELISA kit, Cat # abx254657, Abbexa), IL-6 (Mouse IL-6 Duoset ELISA, Cat # DY406, R&D Systems), IL-1β (Mouse IL-1 beta/IL-1F2 Duoset ELISA, Cat #DY401, R&D Systems), TNF-α (Mouse TNF-alpha DuoSet ELISA, Cat # DY410, R&D Systems), CCL-2 (Mouse CCL-2 DuoSet ELISA, Cat # DY479, R&D Systems), CXCL-9 (Mouse CXCL9 DuoSet ELISA, Cat # DY492, R&D Systems), CCL20 (Mouse CCL-20 DuoSet ELISA, Cat # DY760, R&D Systems) concentrations were measured by sandwich ELISA according to the manufacturer’s protocols.

### Statistical analysis

Data were analyzed using GraphPad Prism software, version v10.1.0 (GraphPad software). Data are presented as mean ± SEM. The number of biological replicates used are indicated in the corresponding figure legend. Normality of the distributed data was tested by Shapiro-Wilk test. Groups were compared using an unpaired t-test or one-way ANOVA with Geisser-Greenhouse correction followed by a Holm-Šídák multiple comparison test in the case of normal distribution. In absence of normal distribution, a Friedman test followed by Dunn’s multiple comparison test was performed. P value of < 0.05 was considered significant throughout.

## Results

MMP-9 deficiency is associated with reduced recovery of myelin markers during remyelination phase in LPC-treated brain slices through microglia/macrophage-mediated mechanisms.

**Fig. 1:**
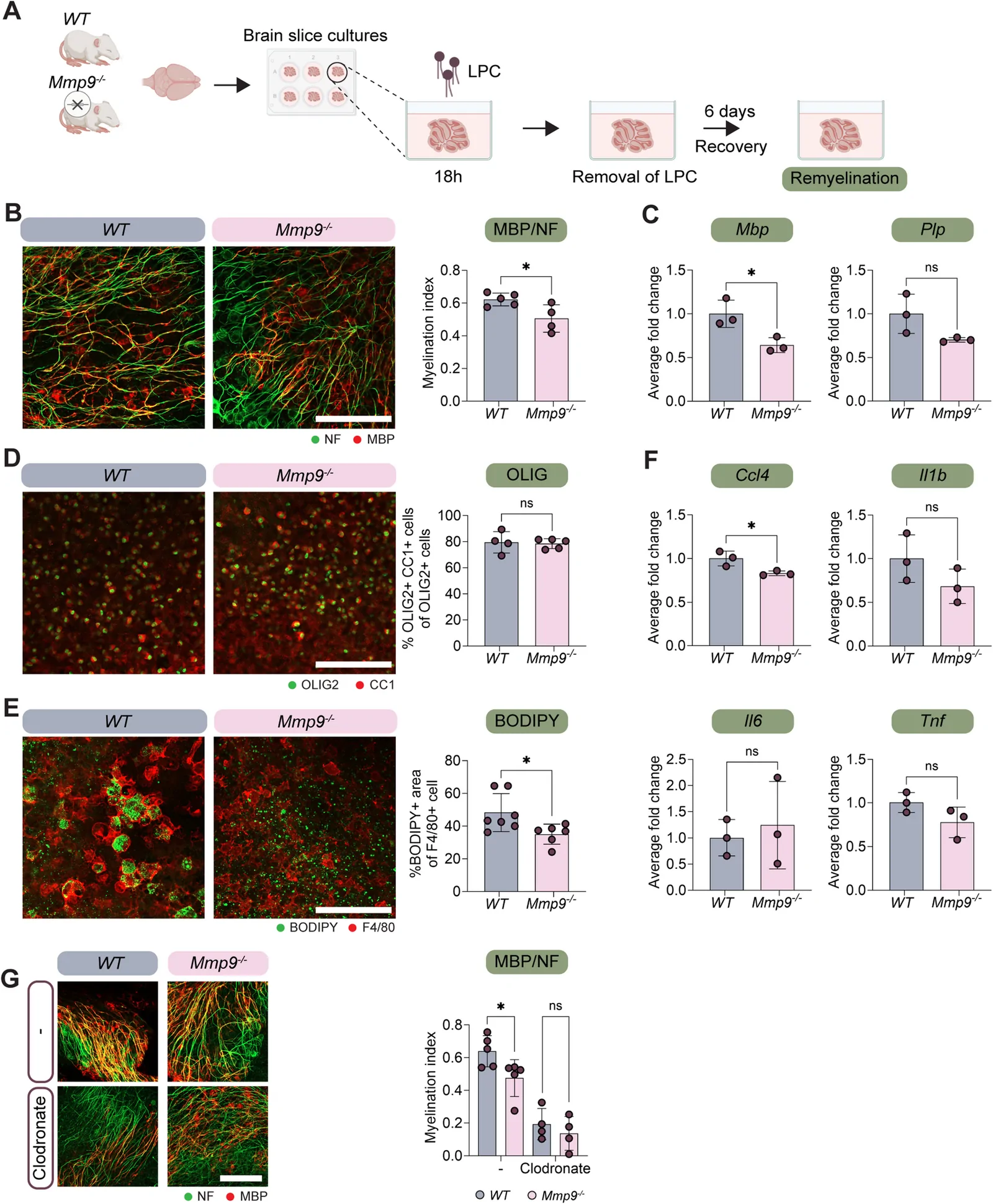
MMP-9 deficiency impairs remyelination in LPC-treated brain slices through microglia/macrophage-mediated mechanisms. (**A**) Schematic overview of the experimental design. Brain slices were isolated from p9-10 *WT* and *Mmp9*^*−/−*^ mouse pups, demyelinated with LPC for 18 h and recovered in basal medium for 6 days after which remyelination was assessed. (**B**) Representative immunofluorescence images of remyelinated brain slices stained for MBP (red) and NF (green) and quantification of MBP + NF+ axons out of total NF+ axons in LPC-treated *WT* and *Mmp9*^*−/−*^ brain slices (at least *n* = 4 mice/group of 2 independent experiments). The scale bar represents 100 μm. Bars represent the mean, symbols represent individual brain slices, and error bars indicate the standard error of mean. P values less than 0.05 were considered significant (* *p* < 0.05). (**C**) Quantification of *Mbp* and *Plp* mRNA levels after remyelination LPC-treated *WT* and *Mmp9*^*−/−*^ brain slices by qRT-PCR (data were normalized to 18 S mRNA levels) (*n* = 3 mice/group of 2 independent experiments). Bars represent the mean, symbols represent individual brain slices, and error bars indicate the standard error of mean. P values less than 0.05 were considered significant (* *p* < 0.05; ns non-significant). (**D**) Representative immunofluorescence images of remyelinated brain slices stained for OLIG2 (red) and CC1 (green) and quantification of OLIG2 + CC1+ cells within the OLIG2 + cell population in LPC-treated *WT* and *Mmp9*^*−/−*^ brain slices (at least *n* = 4 mice/group of 2 independent experiments.). The scale bar represents 100 μm. Bars represent the mean, symbols represent individual brain slices, and error bars indicate the standard error of mean. P values less than 0.05 were considered significant (ns non-significant). (**E**) Representative immunofluorescence images of remyelinated brain slices stained for F40/80 (red) and BODIPY (green) and quantification of area of lipid droplets in total brain slice of LPC-treated *WT* and *Mmp9*^*−/−*^ (at least *n* = 6 mice/group of 2 independent experiments.). The scale bar represents 100 μm. Bars represent the mean, symbols represent individual brain slices, and error bars indicate the standard error of mean. P values less than 0.05 were considered significant (* *p* < 0.05; ns non-significant). (**F**) Quantification of *Ccl4*, *Il1b*, *Il6* and *Tnf* mRNA levels after remyelination of LPC-treated *WT* and *Mmp9*^*−/−*^ brain slices by qRT-PCR (data were normalized to 18 S mRNA levels) (*n* = 3 mice/group of 2 independent experiments). Bars represent the mean, symbols represent individual brain slices, and error bars indicate the standard error of mean. P values less than 0.05 were considered significant (* *p* < 0.05; ns non-significant). (**G**) Representative immunofluorescence images of remyelinated brain slices stained for MBP (red) and NF (green) and quantification of MBP + NF+ axons out of total NF+ axons in LPC-treated *WT* and *Mmp9*^*-/-*^ brain slices, upon depletion of microglia/macrophages with clodronate liposomes compared to controls treated with empty liposomes (-) (at least *n* = 4/group of 2 independent experiments). The scale bar represents 100 μm. Bars represent the mean, symbols represent individual brain slices, and error bars indicate the standard error of mean. P values less than 0.05 were considered significant (* *p* < 0.05; ns non-significant). CCL, CC chemokine ligand; IL, interleukin; LPC, lysophosphatidylcholine; MBP, myelin basic protein; MMP, matrix metalloprotease; NF, neurofilament; OLIG, oligodendrocyte; PLP, myelin proteolipid protein; qRT-PCR, quantitative reverse transcription-polymerase chain reaction; TNF, tumor necrosis factor and WT, wild-type

Previously, *Mmp9*^*−/−*^ mice subjected to in vivo LPC demyelination were shown to exhibit impaired remyelination [35]. To validate these findings, we employed ex vivo cerebellar brain slice cultures from *WT* and *Mmp9*^*−/−*^ mice and induced demyelination using LPC (Fig. 1A). Fluorescent staining revealed reduced colocalization of MBP and neurofilament (NF) in *Mmp9*^*−/−*^ slices, indicating impaired axonal remyelination (Fig. 1B). Consistently, *Mmp9*^*−/−*^ slices displayed lower mRNA levels of *Mbp* and *Plp* compared to *WT* following LPC treatment (Fig. 1C). Despite this deficit, the abundance of OLIG2+ CC1+ mature oligodendrocytes was comparable between genotypes, suggesting that the remyelination impairment was not due to defective OPC differentiation (Fig. 1D). Interestingly, *Mmp9* deficiency also reduced intracellular lipid accumulation in microglia, as evidenced by a lower percentage BODIPY+ area in F4/80+ cells in *Mmp9*^*−/−*^ slices relative to *WT* (Fig. 1E). Moreover, the absence of *Mmp9* decreased expression of the inflammatory chemokine *Ccl4* without affecting the expression of proinflammatory cytokines such as *Il1b*,* Il6* and *Tnf* (Fig. 1F, Suppl. Fig. S1), and neurotrophic factor expression (Suppl. Fig. S1).

Given the observed reduction in lipid content within F4/80+ microglia and our previous findings implicating intracellular lipid accumulation as a key regulator of microglial function [39, 40], we next investigated the contribution of these phagocytes to the remyelination deficit in *Mmp9*^*−/−*^ slices. To this end, we employed a cell-depletion strategy using clodronate liposomes, previously shown by us to selectively deplete microglia in brain slices [41, 42]. As expected, empty liposome-treated slices recapitulated the remyelination deficit in *Mmp9*^*−/−*^ mice relative to WT. However, this effect was abolished following microglia depletion using clodronate liposomes (Fig. 1G), indicating a key role for these phagocytes in the observed phenotype. Notably, microglia depletion did not alter OLIG2+ CC1+ oligodendrocyte abundance in LPC-treated slices of either genotype (Suppl. Fig. S1), suggesting that the impaired remyelination may result from a reduced number of axons myelinated per oligodendrocyte rather than from defective oligodendrocyte differentiation.

Collectively, these findings demonstrate that MMP-9 deficiency impairs remyelination and reduces microglia lipid load, underscoring a critical role for MMP-9 in microglia in facilitating myelin repair.

MMP-9 deficiency spares demyelination and remyelination but alters phagocyte lipid accumulation in the CPZ model.

**Fig. 2:**
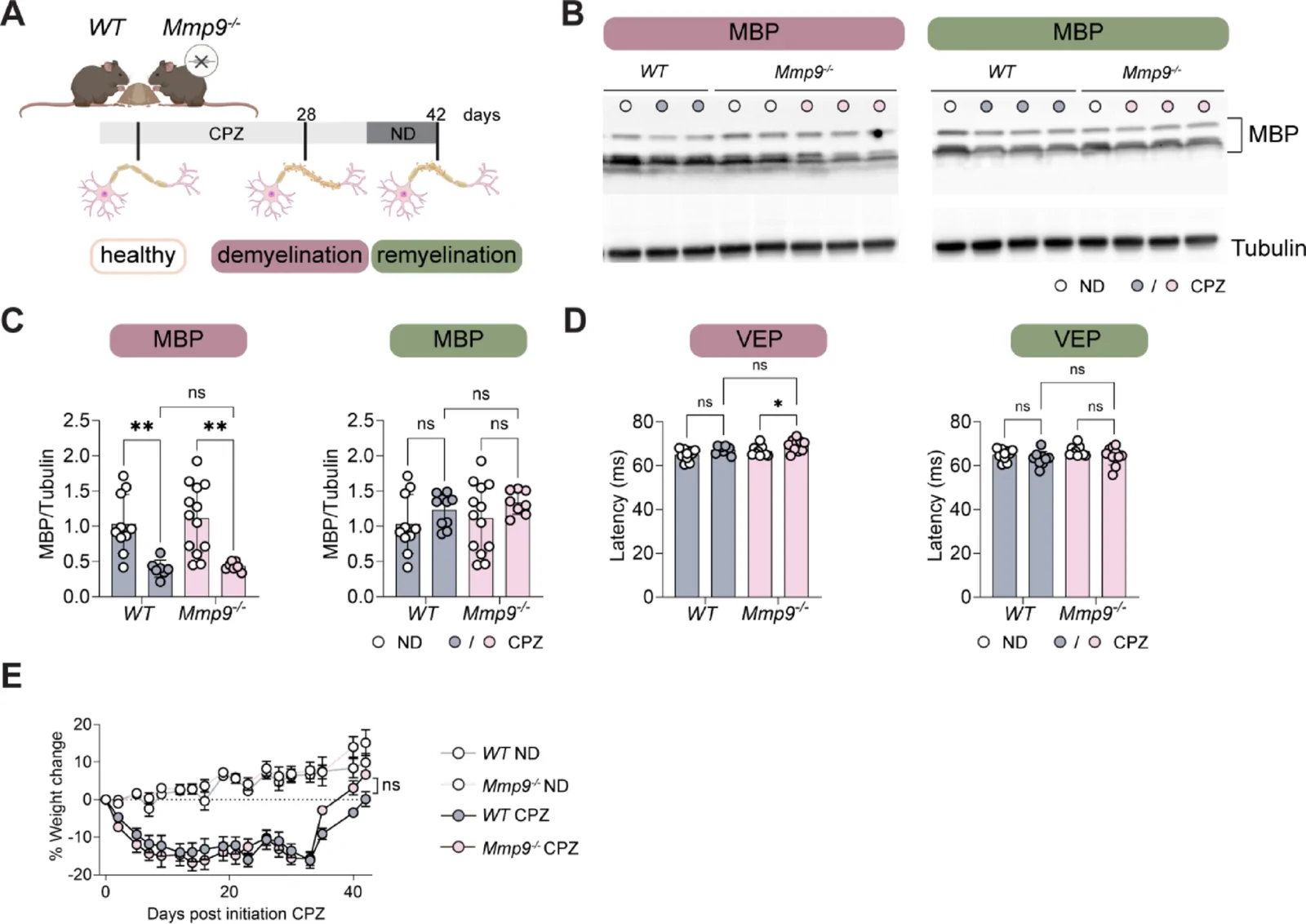
MMP-9 does not influence demyelination and remyelination processes in the corpus callosum upon CPZ diet. (**A**) Schematic overview of the experimental design. Demyelination was induced by feeding *WT* and *Mmp9*^*−/−*^ mice with CPZ for a total of five weeks. Upon four weeks mice of CPZ diet were sacrificed and analysed to study processes associated with demyelination (full purple boxes). Mice in the control group were treated with ND without CPZ. To study remyelination, mice received an additional week of ND upon the CPZ diet, before being sacrificed (full green boxes). (**B**) and (**C**) Quantification of MBP in brain lysates of *WT* and *Mmp9*^*−/−*^ mice during demyelination and remyelination compared to ND controls by Western blot analysis (data were normalized to tubulin) (at least *n* = 7 mice/group of 3 independent experiments). Bars represent the mean, symbols represent individual mice, and error bars indicate the standard error of mean. P values less than 0.05 were considered significant (** *p* < 0.01; ns non-significant). (**D**) Latency times from VEPs is given as a measure for fiber myelination (at least *n* = 9 mice/group of 1 independent experiment). Bars represent the mean, symbols represent individual mice, and error bars indicate the standard error of mean. P values less than 0.05 were considered significant (* *p* < 0.05; ns non-significant). (**E**) Percentage weight change of *WT* and *Mmp9*^*−/−*^ mice upon CPZ and ND over time. Symbols represent the average of at least *n* = 8 mice/group of 4 independent experiment, and error bars indicate the standard error of mean. P values less than 0.05 were considered significant (ns non-significant). CC, corpus callosum; CPZ, cuprizone; MBP, myelin basic protein; MMP, matrix metalloprotease; ND, normal diet; VEP, visual evoked potential; *WT*, wild-type

To further investigate the role of MMP-9 in in vivo demyelination and remyelination, we employed the CPZ-induced demyelination model, a well-established experimental setting for studying myelin damage and subsequent repair within the CNS. In this model, the copper chelator CPZ selectively targets mature oligodendrocytes, resulting in extensive demyelination, most prominently within the CC, followed by spontaneous remyelination upon withdrawal of the toxin [37]. We first assessed whether *Mmp9* deficiency influences the extent of CPZ-induced demyelination. *WT* and *Mmp9*^*–/–*^ mice were fed a diet containing 0.3% CPZ for four weeks, corresponding to peak demyelination, and sacrificed at the end of the intoxication period to evaluate myelin integrity (Fig. 2A; purple headers). *Mbp* and *Plp* mRNA and MBP protein levels in the CC were significantly reduced in both *WT* and *Mmp9*^*−/−*^ mice compared to their respective ND controls at day 28 (Fig. 2B, C and Suppl. Fig. S2-3), indicating comparable levels of demyelination across genotypes. *Mmp9* deficiency did not affect intracellular lipid accumulation in microglia, reflected by ORO reactivity in the CC of *Mmp9*^*−/−*^ mice (Suppl. Fig. S3). To evaluate remyelination, *WT* and *Mmp9*^*−/−*^ mice were returned to ND for one week following CPZ intoxication (Fig. 2A; green headers). *Mbp* and *Plp* mRNA and MBP protein levels (Fig. 2B-C and Suppl. Fig. S2-3), as well as lipid content (Suppl. Fig. S3), recovered to levels comparable to ND controls in both genotypes, demonstrating remyelination. *Mmp9*^*−/−*^ mice exhibited remyelination equivalent to *WT* mice, indicating that MMP-9 is not required for spontaneous myelin repair in this model.

In addition, we observed no differences in ORO lipid load in the CC of *Mmp9*^*−/−*^ mice, suggesting that MMP-9 does not modulate the formation of foamy microglia during CPZ-induced demyelination (Suppl. Fig. S3).

We next examined whether MMP‑9 influences VEP latency in the CPZ model. During the final week of demyelination, CPZ‑treated mice displayed prolonged latency compared to ND controls, consistent with impaired myelination. MMP-9 deficiency did not alter VEP latency during this phase (Fig. 2D). During remyelination, latency values returned to control levels in CPZ‑treated mice of both genotypes, again indicating MMP‑9 does not affect functional recovery of conduction velocity.

Because body weight loss has been reported to correlate with the severity of CPZ-induced demyelination [43], we monitored the body weight of *Mmp9*^*−/−*^ mice throughout CPZ exposure. As expected, CPZ-intoxicated mice exhibited significant weight loss compared to ND-fed controls. However, no significant difference in weight loss was observed between *WT* and *Mmp9*^*−/−*^ mice (Fig. 2E).

Thus, these findings indicate that MMP-9 does not directly influence the extent of demyelination or remyelination in the CPZ-induced demyelination model, although it may modulate microglial lipid handling.

Number of microglia/macrophages is increased in corpus callosum of *Mmp9*^*-/-*^ CPZ mice compared to *WT* CPZ mice.

**Fig. 3:**
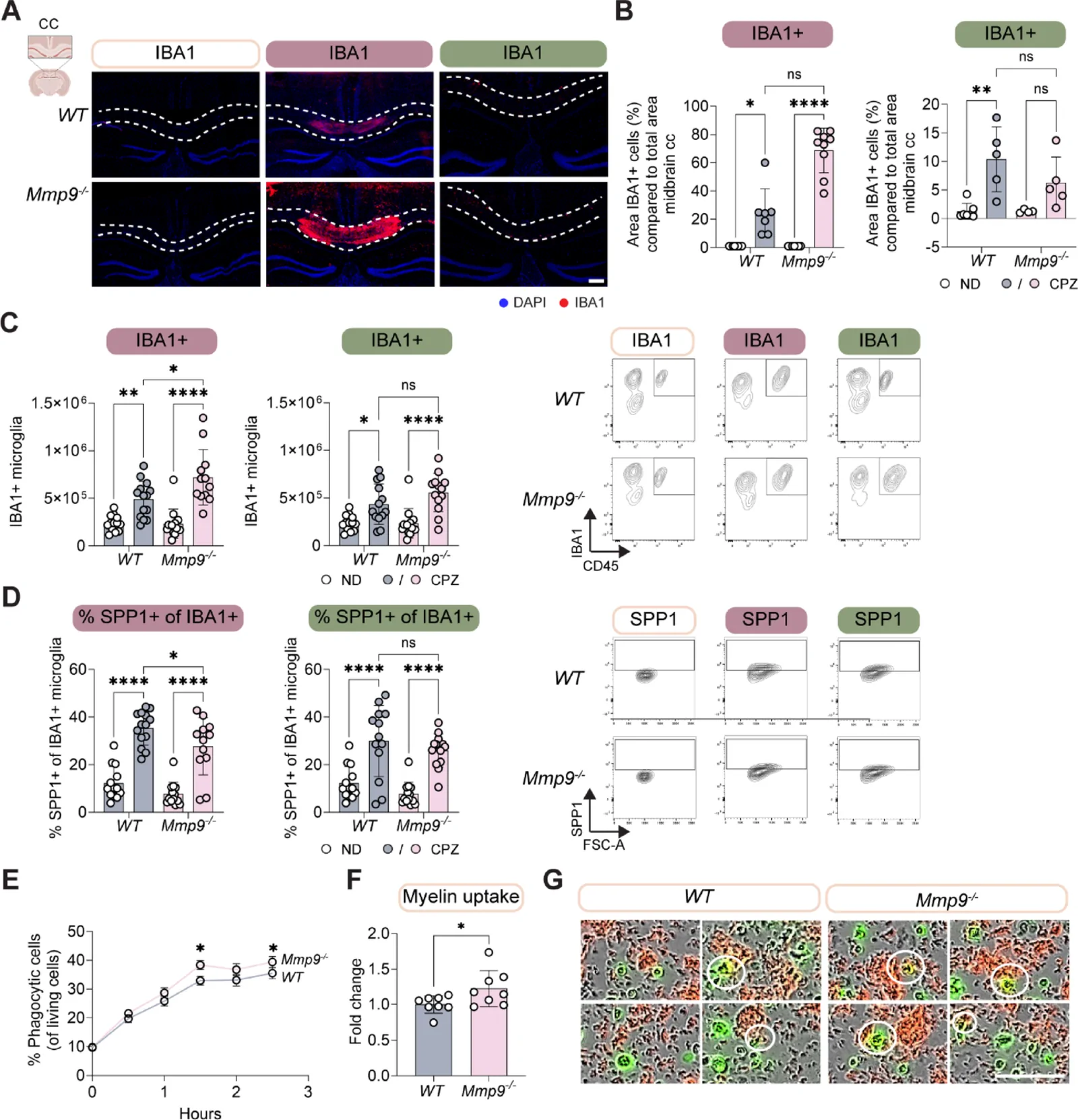
IBA1+ positive cells are increasingly present in corpus callosum of *Mmp9*^***−/−***^ CPZ mice compared to *WT* CPZ mice during demyelination. CPZ-induced demyelination model was induced in *WT* (grey bars) and *Mmp9*^*−/−*^ mice (pink bars) as explained in Fig. 2A. Mice were either sacrificed after four weeks of CPZ treatment (indicated by solid purple boxes) to assess demyelination, or after five weeks of CPZ followed by one week of normal diet (solid green boxes) to evaluate remyelination. (**A**) Representative immunofluorescent images of IBA1 staining (red) on brain sections of *WT* and *Mmp9*^*−/−*^ mice upon CPZ and ND, indicating activated microglia/macrophages. Cell nuclei are visualised by Hoechst 33,342 staining (DAPI, blue). White dashed line indicates CC. The scale bar represents 500 μm. (**B**) Quantification of area IBA1+ cells in midbrain CC compared to the area of total midbrain CC (%) of *WT* and *Mmp9*^*−/−*^ mice upon CPZ and ND (at least *n* = 10 mice/group of 3 independent experiments). Bars represent the mean, symbols represent individual mice, and error bars indicate the standard error of mean. P values less than 0.05 were considered significant (* *p* < 0.05; ** *p* < 0.01; *** *p* < 0.001; ns non-significant). (**C**) Representative plots and quantification of absolute number of IBA1 + cells in CC of *WT* and *Mmp9*^*−/−*^ mice upon CPZ and ND, assessed by flow cytometry (at least *n* = 10 mice/group of 2 independent experiments). Bars represent the mean, symbols represent individual mice, and error bars indicate the standard error of mean. P values less than 0.05 were considered significant (** *p* < 0.01; *** *p* < 0.001; ns non-significant). (**D**) Representative plots and quantification of percentage SPP1+ of IBA1+ cells in CC of *WT* and *Mmp9*^*−/−*^ mice upon CPZ and ND, assessed by flow cytometry (at least *n* = 10 mice/group of 2 independent experiments). Bars represent the mean, symbols represent individual mice, and error bars indicate the standard error of mean. P values less than 0.05 were considered significant (* *p* < 0.05; **** *p* < 0.0001; ns non-significant). (**E**) Percentage of naïve *WT* and *Mmp9*^*−/−*^ mice microglia that phagocytosed fluorescently-labelled myelin debris over time. Symbols represent the average of *n* = 8 individual mice of 2 independent experiments, and error bars indicate the standard error of mean. P values less than 0.05 were considered significant (* *p* < 0.05). (**F**) Quantification of naïve *WT* and *Mmp9*^*−/−*^ microglia phagocytosing fluorescently-labelled myelin debris after 1.5 h of incubation (= 10 mice/group of 2 independent experiments). Bars represent the mean, symbols represent individual mice, and error bars indicate the standard error of mean. P values less than 0.05 were considered significant (* *p* < 0.05). (**G**) Representative bright field images of naïve *WT* and *Mmp9*^*−/−*^ microglia (green) phagocytosing fluorescently-labelled myelin debris (red) after 1.5 h of incubation. Phagocytosing cells are yellow and are encircled in white. The scale bar represents 50 μm. CC, corpus callosum; CPZ, cuprizone; MMP, matrix metalloprotease; ND, normal diet; *WT*, wild-type

CPZ intoxication induces apoptotic loss of oligodendrocytes, leading to demyelination accompanied by microglial activation and astrogliosis, which together contribute to an inflammatory environment [37]. To determine changes in microglia/macrophage abundance, we compared IBA1 levels, a widely used marker of CNS-associated myeloid cells, between *WT* CPZ and *Mmp9*^*−/−*^ CPZ mice [37, 44]. Immunofluorescent staining of IBA1, revealed an increase in IBA1+ area within the CC of CPZ-treated mice (Fig. 3A-B). Notably, this increase was more pronounced in the midbrain CC of *Mmp9*^*−/−*^ mice compared to *WT* mice after 28 days of CPZ exposure. Flow cytometry further confirmed a higher number of IBA1+ cells (CD45+ IBA1+) [45] in *Mmp9*^*−/−*^ CPZ mice relative to *WT* CPZ mice (Fig. 3C; gating strategy can be found in Suppl. Fig. S4). After one week of CPZ withdrawal, IBA1+ cell numbers remained elevated in both genotypes compared to ND controls, although no significant differences persisted between *WT* and *Mmp9*^*−/−*^ mice at this stage.

Microglia are highly dynamic cells whose phenotype rapidly adapts to environmental cues [45, 46]. To assess whether MMP-9 influences the presence of a microglial subset associated with demyelination (SPP1+ IBA1+) [46], we quantified SPP1 expression in microglia/macrophages within the CC after 28 days of CPZ treatment. The proportion of SPP1+ microglia/macrophages was significantly increased in CPZ-treated mice compared to ND controls (Fig. 3D). Interestingly, *Mmp9*^*–/–*^ mice displayed a significant reduction in the percentage of SPP1+ microglia/macrophages relative to *WT* CPZ mice, suggesting that MMP-9 contributes to the induction of this reactive microglial phenotype, particularly at earlier stages, although this effect appears to diminish during remyelination. SPP1 has been implicated in opsonization, phagocytosis, proliferation and lipid metabolism in microglia, making this shift particularly relevant.

Since efficient remyelination requires microglial clearance of myelin debris following demyelinating injury [47], and given the increased microglial numbers in *Mmp9*^*−/−*^ mice, we hypothesized that MMP-9 may influence microglial phagocytic activity. To test this hypothesis, we isolated primary *WT* and *Mmp9*^*−/−*^ microglia and incubated them ex vivo with pHrodo-labeled myelin debris. pHrodo fluorescence increases upon internalisation into acidic phagosomes. After 1.5 h, a higher percentage of *Mmp9*^*−/−*^ microglia had phagocytosed myelin debris compared to *WT* microglia, indicating enhanced phagocytic capacity or impaired processing/degradation of internalized myelin debris in the absence of MMP-9 (Fig. 3E–G).

Collectively, these findings indicate that MMP-9 deficiency increases microglial numbers in the midbrain CC of CPZ-treated mice while reducing the proportion of SPP1+ microglia/macrophages associated with altered myelin handling, highlighting a modulatory role for MMP-9 in microglial phenotype.

Selective chemokine modulation in CSF by MMP-9 does not translate to altered immune cell recruitment in CPZ-treated mice.

**Fig. 4:**
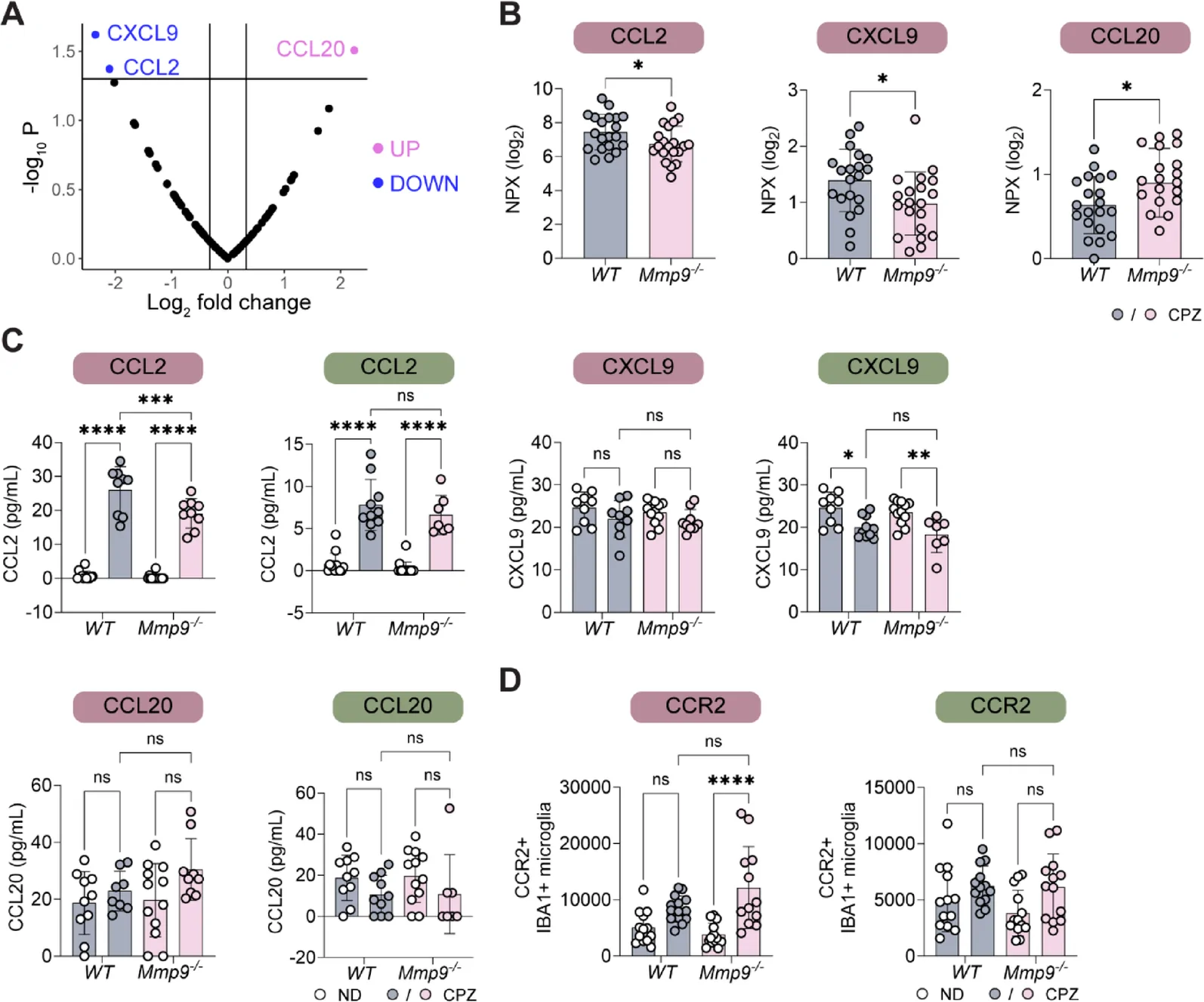
MMP-9 deficiency alters chemokines in CSF and corpus callosum of CPZ mice. CPZ-induced demyelination model was induced in *WT* (grey bars) and *Mmp9*^*−/−*^ mice (pink bars) as explained in Fig. 2A. Mice were either sacrificed after four weeks of CPZ treatment (indicated by solid purple boxes) to assess demyelination, or after five weeks of CPZ followed by one week of normal diet (solid green boxes) to evaluate remyelination. (**A**) Volcano plot of differentially expressed proteins in CSF of *WT* CPZ and *Mmp9*^*−/−*^ CPZ mice, as assessed by Olink^®^ proteomics. Differentially expression protein data points indicate differentially-expressed proteins with uncorrected *p* < 0.05. Black data points indicate proteins with insignificant differences, blue indicates downregulated and pink indicates upregulated. (**B**) Relative protein concentrations of CCL2, CXCL9 and CCL20 in CSF of *WT* CPZ and *Mmp9*^*−/−*^ CPZ mice were quantified in terms of log base-two NPX values, as assessed by Olink^®^ proteomics (*n* = 20 mice/group of 4 independent experiments). Bars represent the mean, symbols represent individual mice, and error bars indicate the standard error of mean. P values less than 0.05 were considered significant (* *p* < 0.05). (**C**) Protein concentrations CCL2, CXCL9 and CCL20 in CC lysate of *WT* and *Mmp9*^*−/−*^ upon CPZ and ND by ELISA per 500 ng protein (at least *n* = 7 mice/group of 3 independent experiments). Bars represent the mean, symbols represent individual mice, and error bars indicate the standard error of mean. P values less than 0.05 were considered significant (* *p* < 0.05; ** *p* < 0.01; *** *p* < 0.001; **** *P* < 0.0001; ns non-significant). (**D**) Number of CCR2 + IBA1+ microglia of *WT* and *Mmp9*^*−/−*^ in CC of *WT* and *Mmp9*^*−/−*^ mice upon CPZ and ND, assessed by flow cytometry (at least *n* = 9/group of 2 individual experiments). Bars represent the mean, symbols represent individual mice, and error bars indicate the standard error of mean. P values less than 0.05 were considered significant (** *p* < 0.01; *** *p* < 0.001; ns non-significant). CC, corpus callosum; CPZ, cuprizone; CSF, cerebrospinal fluid; MMP, matrix metalloprotease; ND, normal diet; NPX, normalized protein expression; *WT*, wild-type

Given that MMP-9 influences microglia/macrophage numbers in the brain of CPZ-treated mice, we next investigated whether MMP-9 modulates inflammatory pathways in the CNS. To this end, we employed a highly sensitive proximity-extension assay combined with qPCR to quantify 92 proteins in the CSF of *WT* and *Mmp9*^*−/−*^ mice during demyelination. Among the 92 proteins analysed, three chemokines were differentially expressed in *Mmp9*^*−/−*^ CPZ mice compared to *WT* CPZ mice (Fig. 4A). Specifically, CCL2 and CXCL9 levels were downregulated, while CCL20 was upregulated in the CSF of *Mmp9*^*−/−*^ mice (Fig. 4B).

To determine whether these chemokine alterations were also present in the brain parenchyma, we analysed their expression in the CC after 28 days of CPZ treatment. Consistent with the CSF data, CCL2 levels were reduced in *Mmp9*^*−/−*^ mouse brains compared to *WT*. However, no significant differences in CXCL9 and CCL20 levels were observed in brain lysates. During the remyelination phase, levels of CCL2, CXCL9 and CCL20 were comparable between *WT* CPZ and *Mmp9*^*−/−*^ CPZ mice (Fig. 4C). Interestingly, an increased number of CCR2-expressing microglia/macrophages in *Mmp9*^*−/−*^ mice were observed during demyelination (Fig. 4D), suggesting that CCR2 signalling remains functionally relevant despite the observed reduction in total CCL2 levels. MMP-9 is known to modulate chemokine activity and availability through selective proteolytic cleavage [48, 49]. Indeed, we observed that active MMP-9 proteolytically processes recombinant CCL2 (Suppl. Fig. S5). Importantly, such processing has been reported to generate truncated CCL2 forms with reduced or antagonistic activity towards CCR2 signalling [48].

Since the chemokines altered in the CSF of *Mmp9*^*−/−*^ mice are primarily involved in the recruitment of activated natural killer (NK) cells and lymphocytes to sites of inflammation and only minor BBB disruption has been reported in the CPZ-induced model [37], we hypothesized that the involvement of these immune cells would be minimal during demyelination and remyelination. Nevertheless, we assessed the infiltration of B cells, NK cells and T cells in the CC of *WT* CPZ and *Mmp9*^*−/−*^ mice using flow cytometry. As expected, no significant differences were observed in the abundance of these NK cells and lymphocyte populations between genotypes (Suppl. Fig S6). Furthermore, expression levels of chemokine receptors on these immune cells were comparable between *WT* CPZ and *Mmp9*^*−/−*^ CPZ mice (Suppl. Fig. S7).

In summary, although MMP-9 deficiency alters chemokine levels in the CSF, specifically increasing CCL20 and decreasing CCL2 and CXCL9, it does not appear to influence the infiltration of B cells, NK cells and T cells into the CC.

### Compensatory MMP regulation and cytokine dynamics in the absence of MMP-9

**Fig. 5:**
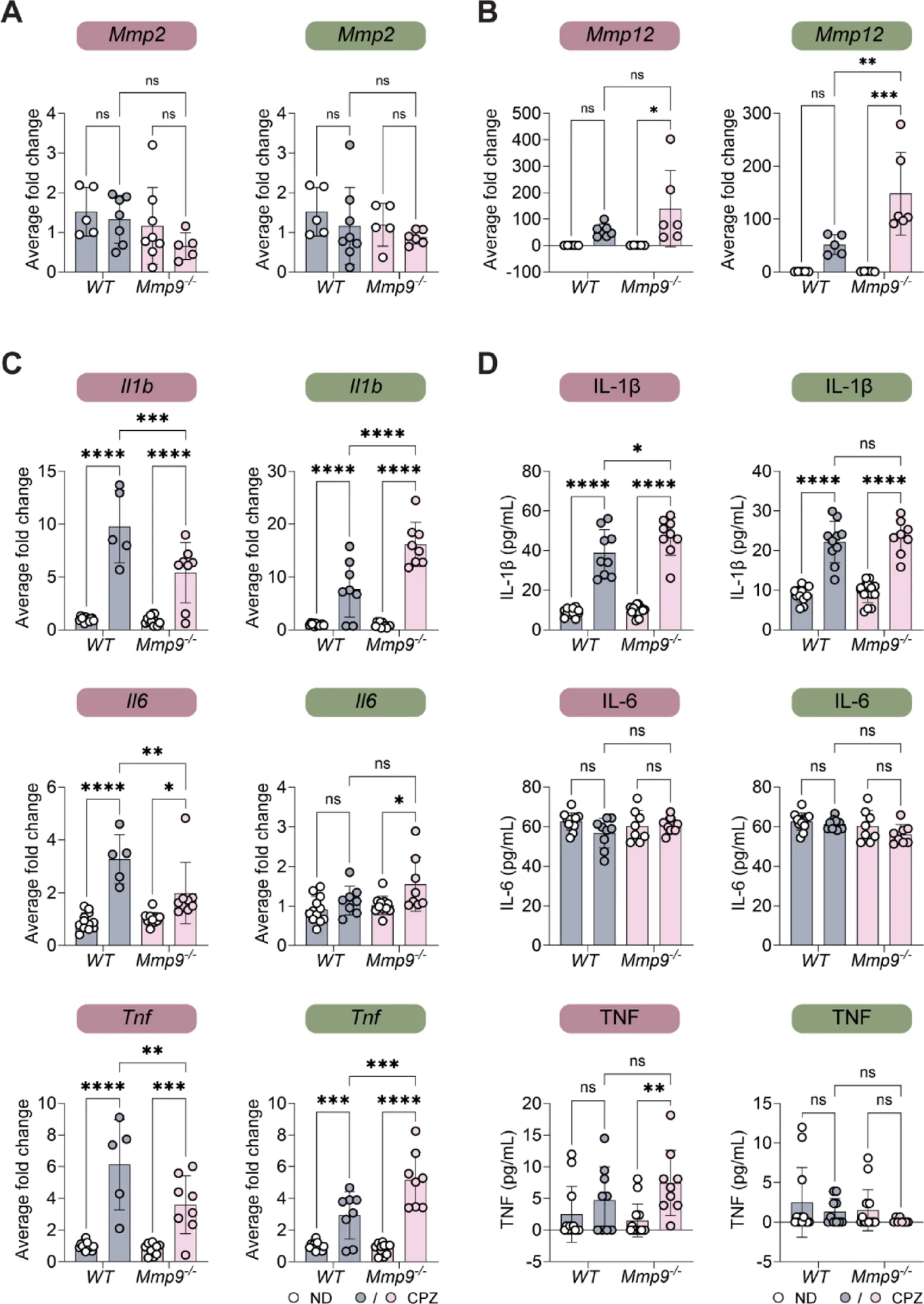
Inflammatory environment in corpus callosum is mildly altered in *Mmp9*^***−/−***^ CPZ mice compared to *WT* CPZ mice. CPZ-induced demyelination model was induced in *WT* (grey bars) and *Mmp9*^*−/−*^ mice (pink bars) as explained in Fig. 2A. Mice were either sacrificed after four weeks of CPZ treatment (indicated by solid purple boxes) to assess demyelination, or after five weeks of CPZ followed by one week of normal diet (solid green boxes) to evaluate remyelination. (**A**) Quantification of *Mmp2* mRNA levels of in CC of *WT* and *Mmp9*^*−/−*^ upon CPZ and ND by RT-qPCR (data were normalized to *18 S* mRNA levels) (*n* = 5 mice/group of 2 independent experiments). Bars represent the mean, symbols represent individual mice, and error bars indicate the standard error of mean. P values less than 0.05 were considered significant (ns non-significant). (**B**) Quantification of *Mmp12* mRNA levels of in CC of *WT* and *Mmp9*^*−/−*^ upon CPZ and ND by RT-qPCR (data were normalized to *18 S* mRNA levels) (*n* = 5 mice/group of 2 independent experiments). Bars represent the mean, symbols represent individual mice, and error bars indicate the standard error of mean. P values less than 0.05 were considered significant (* *p* < 0.05; ** *p* < 0.01; *** *p* < 0.001; ns non-significant). (**C**) Quantification of *Il1b*, *Il6* and *Tnf* mRNA levels of in CC of *WT* and *Mmp9*^*−/−*^ upon CPZ and ND by RT-qPCR (data were normalized to *18 S* mRNA levels) (*n* = 10 mice/group of 3 independent experiments). Bars represent the mean, symbols represent individual mice, and error bars indicate the standard error of mean. P values less than 0.05 were considered significant (* *p* < 0.05; ** *p* < 0.01; *** *p* < 0.001; **** *p* < 0.0001; ns non-significant). (**D**) Protein concentrations IL-1β, IL-6 and TNF in CC lysate of *WT* and *Mmp9*^*−/−*^ mice upon CPZ and ND by ELISA per 500 ng protein (*n* = 10 mice/group of 3 independent experiments). Bars represent the mean, symbols represent individual mice, and error bars indicate the standard error of mean. P values less than 0.05 were considered significant (* *p* < 0.05; ** *p* < 0.01; **** *p* < 0.0001; ns non-significant). CC, corpus callosum; CPZ, cuprizone; CSF, cerebrospinal fluid; MMP, matrix metalloprotease; ND, normal diet; RT-qPCR, real-time quantitative polymerase chain reaction; TNF, tumor necrosis factor; *WT*, wild-type

As proteases function within interconnected protease networks, where one protease can compensate for the loss of another, we examined whether MMP-9 deficiency alters the expression of other MMPs. Indeed, during demyelination and, even more prominently, during remyelination, we observed increased *Mmp12* mRNA expression in the absence of MMP-9, whereas *Mmp2* mRNA expression levels remained constitutively expressed (Fig. 5A-B).

To further assess how MMP-9 deficiency shapes the inflammatory environment in the CC, we measured the expression of pro-inflammatory cytokines IL-1β, IL-6 and TNF at both the mRNA and protein level. During demyelination and remyelination, mRNA levels of these cytokines were elevated both in *WT* CPZ and *Mmp9*^*−/−*^ CPZ mice compared to ND controls (Fig. 5C). Notably, *Mmp9*^*−/−*^ CPZ mice exhibited lower cytokine mRNA expression levels than *WT* CPZ mice during demyelination, suggestive for a less inflammatory transcriptional profile. Unexpectedly, this trend was not reflected at the protein level (Fig. 5D). In contrast to the mRNA data, IL-1β protein levels were increased in the CC of *Mmp9*^*−/−*^ CPZ mice compared to *WT* CPZ mice during demyelination. Following one week of cuprizone withdrawal, mRNA expression levels *Il1b*, *Il6* and *Tnf* were elevated in *Mmp9*^*−/−*^ CPZ mice compared to *WT* CPZ mice, while protein levels of these cytokines were not significantly different between genotypes (Fig. 5C, D).

In summary, although mRNA cytokine levels of pro-inflammatory mediators were dysregulated during demyelination and remyelination, this was not reflected in their corresponding protein levels. Importantly, the increased expression levels of *Mmp12* suggest a compensatory or redundant role for MMP-9 in the CPZ-induced demyelination model.

## Discussion

MMP-9 is a key contributor to MS pathogenesis, with elevated levels detected in brain lesions [7–10], serum [50], CSF [11, 12, 50] and PBMCs [12–15], where they correlate with disease activity and lesion formation [16–18]. In the EAE model, MMP-9 disrupts the BBB thereby promoting the peripheral immune cell infiltration into the CNS and exacerbating disease progression [19, 21–23]. Although MMP-9 can degrade MBP in vitro [33], its precise contribution to demyelination and remyelination remains unclear. To address this, we used both ex vivo LPC-treated *Mmp9*^*−/−*^ brain slices and in vivo CPZ-treated *Mmp9*^*−/−*^ mice and compared them to *WT* mice, to examine the role of MMP-9 in myelin loss and repair. While constitutive knockout models present limitations, they remain widely used to assess the overall contribution of a given factor in demyelination and repair processes.

We initially investigated the involvement of MMP-9 in demyelination and remyelination using the ex vivo LPC model, as it enables rapid assessment of these processes compared to more time-intensive in vivo studies. Following demyelination, LPC-treated *Mmp9*^*−/−*^ brain slices displayed impaired remyelination. Importantly, as demyelination was not quantified at early time points in the LPC model, we cannot exclude that differences in initial lesion severity may contribute to the observed differences in remyelination. Similarly, ex vivo LPC-treated *Mmp9*^*−/−*^ mice showed reduced remyelination [35]. Notably, this phenotype was abolished by microglia depletion, highlighting a central role for microglia/macrophages. Furthermore, *Mmp9* deficiency reduced intracellular lipid accumulation in microglia. Together, these findings indicate that MMP-9 deficiency impairs remyelination through microglia/macrophage-dependent mechanisms.

To further investigate the role of MMP-9 in vivo, we employed the CPZ-induced demyelination model. The induction of demyelination and remyelination was confirmed by MBP expression levels and VEP latency time. After four weeks of CPZ treatment, both *WT* and *Mmp9*^*−/−*^ mice exhibited reduced MBP protein levels, indicating effective demyelination. One week after CPZ withdrawal, MBP protein levels returned to baseline, consistent with remyelination. Unexpectedly, no significant differences in MBP protein expression or VEP latency times were observed between *WT* and *Mmp9*^*−/−*^ mice during either phase. Although several MMPs, including MMP-2, MMP-7, MMP-9, MMP-10 and MT6-MMP, can degrade myelin proteins such as MBP, myelin-associated glycoprotein and myelin oligodendrocyte glycoprotein in vitro [22, 33, 51–53], our in vivo findings suggest that MMP-9 does not directly contribute to demyelination or remyelination in the CPZ model. Of note, *Mmp9*^*−/−*^ mice showed elevated *Mbp* mRNA levels, and a slight increase in *Plp* mRNA after four weeks of CPZ, potentially reflecting delayed protein upregulation. A recent study similarly reported no effect of MMP-7 deficiency on CPZ-induced demyelination and remyelination [54]. In contrast, MMP-9 plays a prominent role in the EAE model, where its absence delays disease onset [38], exacerbates peak disease [55] and prolongs remission [36]. Remyelination requires efficient clearance of damaged myelin and differentiation of new oligodendrocytes. In the LPC model, MMP-9 is expressed by oligodendrocytes in the CC [56, 57], where it promotes process extension, thereby supporting remyelination [58]. MMP-9 also cleaves inhibitory molecules such as IGFBP-6, thereby facilitating oligodendrocyte differentiation [56]. Although, *Mmp9* mRNA was mildly upregulated in the CPZ model [59], other MMPs may play more dominant roles during remyelination [60, 61]. Compensatory upregulation of other MMPs may mask the contribution of MMP-9. Indeed, we observed increased *Mmp12* mRNA expression, suggesting functional compensation.

The apparent discrepancy in the involvement of MMP-9 in demyelination and remyelination across different models likely reflects fundamental variations in lesion type, ECM remodelling requirements, lesion kinetics, and the underlying mechanisms of tissue damage and immune involvement. The ex vivo LPC model induces demyelination in cerebellar tissue from early postnatal (P10) mice, whereas the CPZ model induces diffuse demyelination in the CC of adult mice in vivo. This distinction is important, as the early postnatal brain likely exhibits greater plasticity than the adult brain. LPC-induced lesions are characterized by rapid myelin breakdown and pronounced ECM degradation [62–65], processes in which MMP-9 is likely to play a key role through its ability to remodel ECM components and facilitate tissue reorganization [35]. In addition, LPC lesions are associated with the activation of myeloid cells, further increasing reliance on MMP-9–mediated processes. Myelin debris clearance in this model depends primarily on resident microglia/macrophages, as peripheral macrophage recruitment is limited [66]. In contrast, in vivo CPZ-induced demyelination is primarily driven by oligodendrocyte toxicity resulting from mitochondrial dysfunction and oxidative stress, leading to a more diffuse pathology that is less dependent on active ECM remodelling [64, 65]. This reduced reliance on matrix turnover, together with limited peripheral immune cell infiltration, likely diminishes the functional relevance of MMP-9. Collectively, these findings support the notion that MMP-9 contributes to demyelinating pathology primarily through its roles in ECM remodelling and regulation of microglia/macrophage responses, rather than through direct effects on myelin integrity or repair in toxin-driven demyelination such as the CPZ model. Future studies using inducible or temporally controlled MMP-9 targeting approaches will be essential to disentangle its specific roles during demyelination versus remyelination phases.

Microglial recruitment and activation are hallmarks of MS [67] and are well recapitulated in the CPZ-induced demyelination model [68–70]. Consistent with this, we observed an increased number IBA1 + cells in CPZ-treated mice. Interestingly, *Mmp9*^*−/−*^ mice exhibited an even higher density of IBA1 + cells in the CC than *WT* mice, suggesting enhanced microglial recruitment in absence of MMP-9. Microglia adopt diverse activation states during demyelination, ranging from proinflammatory (SPP1, PADI2, APoe) to pro-reparative (CD163, CD68, CD74, FTL, MSR1) profiles [46, 71]. Particularly, SPP1 (OPN)-expressing microglia are associated with neurodegeneration [72]. We observed increased SPP1 expression on IBA1+ cells during CPZ-induced demyelination, which persisted into remyelination. Importantly, *Mmp9*^*−/−*^ mice displayed a reduced proportion of SPP1+ IBA1+ cells, suggesting attenuation of a proinflammatory microglial phenotype. This aligns with the elevated *Mbp* mRNA expression levels in *Mmp9*^*−/−*^ mice, potentially reflecting a less severe demyelinating environment. Our ex vivo LPC findings also pointed to a microglia/macrophage-dependent effect on remyelination. Future studies incorporating additional markers and high-resolution morphological profiling will be required to further characterize functional activation states within this population. To further assess microglial function, we evaluated phagocytic capacity in naïve *WT* and *Mmp9*^*−/−*^ mice. Unexpectedly, MMP-9 deficient microglia ingested more fluorescently-labelled myelin, which may reflect either enhanced phagocytosis of myelin debris or impaired processing and degradation of internalized debris. Future studies examining lysosomal activity, degradation kinetics, and intracellular trafficking of myelin debris will be required to discriminate between these possibilities.

Proteomics analysis using Olink revealed that MMP-9 deficiency primarily affects chemokine expression. Among the 92 proteins assessed, levels of CCL2, CXCL9 and CCL20 were altered in the CSF of CPZ-treated *Mmp9*^*−/−*^ mice compared to *WT* controls. Notably, CCL2 levels were reduced in both CSF and CC of *Mmp9*^*−/−*^ mice, coinciding with increased microglial numbers. This may reflect local consumption [73], promoting microglia recruitment in *Mmp9*^*−/−*^ mice. Of note, MMP-9-mediated proteolytic processing of CCL2 may contribute to this apparent discrepancy, as cleavage of CCL2 can generate antagonistic forms that reduce CCR2 signalling activity [48, 49] despite unchanged or increased total protein levels. Previous studies show that CCL2 ablation in the CC reduces microglia presence and delays demyelination [69, 70]. However, despite increased microglia in *Mmp9*^*−/−*^ mice, demyelination was not delayed. CPZ-treated *Mmp9*^*−/−*^ mice showed increased number of CCR2-expressing microglia/macrophages during demyelination, suggesting that CCR2 signalling remains functionally relevant despite the observed reduction in total CCL2 levels. We also observed decreased CXCL9 and increased CCL20 levels in the CSF of *Mmp9*^*−/−*^ mice. These chemokines are involved in the recruitment of lymphocytes and NK cells into the CNS [74–76], and CCL20 in particular has been associated with disease severity and CCR6+ T cell entry into the CNS in inflammatory models such as EAE [77]. However, its role appears to be highly context-dependent and less prominent in toxin-induced demyelination models. In the CPZ model, which is largely driven by oligodendrocyte toxicity and characterized by limited peripheral immune cell infiltration, we did not observe an induction of CCL20 expression upon demyelination. This suggests that CCL20 is unlikely to play a central role in driving pathology in this setting. In agreement, proteomic analysis of the CSF of CPZ-intoxicated mice did not reveal increased CCL20 levels [78]. Moreover, despite the modulation of CCL20 in *Mmp9*^*−/−*^ mice, these changes were not reflected in the CC, indicating a localized and context-dependent effect. This interpretation is further supported by the absence of differences in B, T, and NK cell infiltration in the corpus callosum between WT and *Mmp9*^*−/−*^ mice, consistent with the largely intact BBB in the CPZ model [79]. Together, these findings indicate that MMP-9–dependent regulation of chemokines such as CCL20 does not necessarily translate into functional immune cell recruitment in models with limited peripheral involvement. Rather, this modulation may reflect broader changes in inflammatory signalling. Future studies will be required to elucidate the precise mechanisms linking MMP-9 activity to chemokine regulation across distinct neuroinflammatory contexts.

To assess the inflammatory environment in the CC, we measured cytokine expression during CPZ-induced demyelination. Consistent with previous reports [80–82], CPZ treatment in *WT* mice led to increased protein levels of IL-1β, TNF, with a modest rise in IL-6. Interestingly, *Mmp9*^*−/−*^ mice showed elevated IL-1β protein levels in the CC, potentially reflecting increased microglial numbers, as microglia and astrocytes are the primary producers of IL-1β in the CPZ-induced demyelination model [83]. Although *Mmp9*^*−/−*^ mice exhibited altered chemokine levels and increased microglial presence, these changes did not substantially modify the overall inflammatory profile. Lastly, our data point towards a role for MMP-9 in modulating microglial myelin handling and lipid accumulation. Notably, the different assay systems used in this study capture distinct aspects of microglial function, including lipid storage in situ and acute myelin uptake under ex vivo conditions. Accordingly, our study does not allow us to distinguish whether MMP-9 primarily affects myelin uptake, lysosomal degradation, or lipid clearance. Instead, our data suggest that MMP-9 influences the broader handling of myelin debris by microglia/macrophages during demyelination, rather than defining a specific mechanistic step within the phagocytic pathway.

In conclusion, our findings indicate that while MMP-9 impairs remyelination in the ex vivo LPC model, this effect appears to be redundant in the more complex in vivo CPZ model. Nonetheless, MMP-9 deficiency was associated with increased microglia/macrophage numbers in the CC of CPZ-intoxicated mice, as well as reduced lipid accumulation and altered myelin handling in the ex vivo LPC model, pointing to a critical role for MMP-9 in neuroinflammation through the modulation of microglia/macrophage responses. A deeper understanding of the distinct function of individual MMPs will be essential for the developing targeted therapeutic strategies aimed at modifying the lesion environment and promoting remyelination in MS.

## Limitations of the study

A limitation of this study is that several methodological constraints may influence the interpretation of our findings. Future studies using inducible or temporally controlled MMP-9 targeting approaches will be essential to disentangle its specific roles during demyelination versus remyelination phases, as constitutive knockout models, while widely used, not allow phase-specific resolution of function.

In addition, because demyelination was not quantified at early time points in the LPC model, we cannot exclude that differences in initial lesion severity contributed to the observed differences in remyelination. Our depletion experiments in slice cultures also present limitations, as clodronate liposomes are not entirely specific to microglia and may affect other cell types. Although we did not observe evidence for major oligodendrocyte loss, indirect effects on tissue homeostasis cannot be fully ruled out.

Furthermore, we did not employ fate-mapping or lineage-tracing approaches to definitively determine the origin of myeloid cell populations, preventing unequivocal discrimination between resident microglia and infiltrating peripheral macrophages, a distinction further complicated by their phenotypic convergence under inflammatory conditions. Accordingly, the term “microglia/macrophages” is used to describe the CD45low CD11b+ IBA1+ population identified in our analyses, which is enriched for resident microglia but may include phenotypically similar infiltrating cells. Finally, in the CPZ model, demyelination and remyelination were assessed using MBP protein levels and VEP latency, but the lack of quantification of OPC numbers and absence of ultrastructural analysis of myelin sheath thickness (e.g., by transmission electron microscopy) limit the depth of evaluation of myelin dynamics.

## Supplementary Information

Below is the link to the electronic supplementary material.

Supplementary Material 1

## Data Availability

All data supporting the findings of this study are available within the paper and its Supplementary Information.
