# Supplementary material for: Matrix metalloprotease-9 modulates microglial/macrophage responses in murine brain demyelination
Source: Acta Neuropathol Commun. 2026 Jul 10;14:202. doi: 10.1186/s40478-026-02371-2 (PMC13637125; doi:10.1186/s40478-026-02371-2)
Supplement: Supplementary file 1 [file 40478_2026_2371_MOESM1_ESM.docx]

**Matrix metalloprotease-9 modulates microglial/macrophage responses in murine brain demyelination**

Eline Bernaerts^1^, Amber De Visscher^1^, Sam Vanherle^2,3^, Marie Rutten^2^, Fran Prenen^4^, Nele Berghmans^1^, Jana Van Broeckhoven^2^, Hanne Meers^1^, Mirre De Bondt^2,5^, Febe Boudry^6^, Sofie Knoops^4^, Tania Mitera^1^, Lien De Somer^1,7^, Jeroen Bogie^2,3^, Patrick Matthys^1^, Jennifer Vandooren^1,6*^

^1^Laboratory of Immunobiology, Rega Institute for Medical Research, KU Leuven, 3000 Leuven, Belgium.

^2^Department of Immunology and Infection, Biomedical Research Institute, Hasselt University; 3590 Diepenbeek, Belgium.

^3^University MS center, 3900 Pelt, Belgium.

^4^Laboratory of Immunoparasitology, Rega Institute for Medical Research, KU Leuven, 3000 Leuven, Belgium.

^5^Laboratory of Molecular Immunology, Rega Institute for Medical Research, KU Leuven, 3000 Leuven, Belgium.

^6^Department of Microbiology, Immunology and Transplantation, KU Leuven Campus Kulak, 8500 Kortrijk, Belgium.

^7^University Hospital Leuven, Laboratory of Pediatric Immunology, 3000 Leuven, Belgium.

* Corresponding author:

Jennifer Vandooren

Department of Microbiology, Immunology and Transplantation

KU Leuven Campus Kulak

8500 Belgium

+32 16 32 22 95

Jennifer.vandooren@kuleuven.be

**Figures**

**Suppl. Fig. S1 *Mmp9* deficiency impairs remyelination in LPC-treated brain slices.** Brain slices were isolated from p9-10 *WT* and *Mmp9^-/-^* mouse pups, demyelinated with LPC for 18 hours and recovered in basal medium for 6 days after which remyelination was assessed. (**A**) Quantification of *Ccl5* and *Ifng* mRNA levels after remyelination of LPC-treated *WT* and *Mmp9^-/-^* brain slices by qRT-PCR (data were normalized to *18S* mRNA levels) (n=3 mice/group of 2 independent experiments). Bars represent the mean, symbols represent individual brain slices, and error bars indicate the standard error of mean. P values less than 0.05 were considered significant (ns non-significant). (**B**) Quantification of *Tgfb* and *Cntf* mRNA levels after remyelination of LPC-treated *WT* and *Mmp9^-/-^* brain slices by qRT-PCR (data were normalized to *18S* mRNA levels) (n=3 mice/group of 2 independent experiments). Bars represent the mean, symbols represent individual brain slices, and error bars indicate the standard error of mean. P values less than 0.05 were considered significant (ns non-significant). (**C**) Representative immunofluorescence images of remyelinated brain slices stained for CC1 (red) and OLIG2 (green) and quantification of quantification of OLIG2+ CC1+ cells within the OLIG2+ cell population in LPC-treated *WT* and Mmp9^-/-^ brain slices, upon depletion of microglia/macrophages with clodronate liposomes compared to controls treated with empty liposomes (-) (at least n=3 mice/group of 2 independent experiments). The scale bar represents 100 µm. Bars represent the mean, symbols represent individual brain slices, and error bars indicate the standard error of mean. P values less than 0.05 were considered significant (ns non-significant). CCL, CC chemokine ligand; IFN, interferon; LPC, lysophosphatidylcholine; MMP, matrix metalloprotease; OLIG, oligodendrocyte; qRT-PCR, quantitative reverse transcription-polymerase chain reaction; TGF, transforming growth factor and *WT*, wild-type.

**Suppl. Fig. S2 Representative pictures of MBP expression in corpus callosum of *Mmp9^-/-^*** **CPZ mice and *WT* CPZ mice.** CPZ-induced demyelination model was induced in *WT* and *Mmp9^-/-^* mice as explained in **Fig. 2A**. Mice were either sacrificed after four weeks of CPZ treatment to assess demyelination, or after five weeks of CPZ followed by one week of normal diet to evaluate remyelination. Western blot of MBP in brain lysates of *WT* and *Mmp9^-/-^* mice during demyelination and remyelination compared to ND controls by Western blot analysis (data were normalized to tubulin) (at least n=7 mice/group of 3 independent experiments). Samples 1-9, *Mmp9^-/-^* CPZ mice assessed at demyelination; Samples 10-17 and 57-63, *Mmp9^-/-^* ND mice, Samples, 18-26, *WT* CPZ mice assessed at demyelination, Samples 27-33 and 44-48, *WT* ND mice; Samples 49-56, *Mmp9^-/-^* CPZ mice assessed at remyelination; Samples 34-43, *WT* CPZ mice assessed at remyelination. CC, corpus callosum; CPZ, cuprizone; MBP, myelin basic protein; MMP, matrix metalloprotease; ND, normal diet; RT-qPCR, real-time quantitative polymerase chain reaction; *WT*, wild-type.

**Suppl. Fig. S3 Reduction of myelin-associated genes in corpus callosum in CPZ model.** CPZ-induced demyelination model was induced in *WT* (grey bars) and *Mmp9^-/-^* mice (pink bars) as explained in **Fig. 2A**. Mice were either sacrificed after four weeks of CPZ treatment (indicated by solid purple boxes) to assess demyelination, or after five weeks of CPZ followed by one week of normal diet (solid green boxes) to evaluate remyelination. (**A**) Quantification of *Mbp* mRNA levels in CC of *WT* and *Mmp9^-/-^* mice upon CPZ and ND by RT-qPCR (data were normalized to *18S* mRNA levels) (at least n=10 mice/group of 3 independent experiments). Bars represent the mean, symbols represent individual mice, and error bars indicate the standard error of mean. P values less than 0.05 were considered significant (* p < 0.05; **** p < 0.0001; ns non-significant). (**B**) Quantification of *Plp* mRNA levels of in CC of *WT* and *Mmp9^-/-^* mice upon CPZ and ND by RT-qPCR (data were normalized to *18S* mRNA levels) (at least n=10 mice/group of 3 independent experiments). Bars represent the mean, symbols represent individual mice, and error bars indicate the standard error of mean. P values less than 0.05 were considered significant (* p < 0.05; **** p < 0.0001; ns non-significant). (**C**) and (**D**) Representative bright field images and quantification of lipids on brain sections of *WT* and *Mmp9^-/-^* upon CPZ and ND, by ORO staining (at least n=3 mice/group of 2 independent experiments). White dashed line indicates CC. The scale bar represents 300 µm. Bars represent the mean, symbols represent individual mice, and error bars indicate the standard error of mean. P values less than 0.05 were considered significant (*** p < 0.001; **** p < 0.0001; ns non-significant). CC, corpus callosum; CPZ, cuprizone; MBP, myelin basic protein; MMP, matrix metalloprotease; ND, normal diet; PLP, myelin phospholipid protein; RT-qPCR, real-time quantitative polymerase chain reaction; *WT*, wild-type.

**Suppl. Fig. S4 Representative gating strategy of CPZ-associated populations in corpus callosum of *Mmp9^-/-^* and *WT* mice.** CPZ-induced demyelination model was induced in *WT* and *Mmp9^-/-^* mice as explained in **Fig. 2A**. Mice were either sacrificed after four weeks of CPZ treatment to assess demyelination, or after five weeks of CPZ followed by one week of ND to evaluate remyelination. CPZ, cuprizone; MMP, matrix metalloprotease; ND, normal diet; NK, natural killer; *WT*, wild-type.

**Suppl. Fig. S5 MMP-9 processing of CCL2.** Proteolytic cleavage of recombinant CCL2 by active MMP-9, assessed by SDS-PAGE. CCL2 was either incubated without (-) or with (+) active MMP-9 (1:100 mole) for two hours at 37°C. CCL, C-C motif chemokine ligand; kDa, kilodalton; MMP, matrix metalloproteinase 9; SDS-PAGE, Sodium dodecyl-sulphate polyacrylamide gel electrophoresis.

**Suppl. Fig. S6 Infiltration of immune cells in corpus callosum of *WT* and *Mmp9^-/-^*** **CPZ mice compared to ND mice.** CPZ-induced demyelination model was induced in *WT* (grey bars) and *Mmp9^-/-^* mice (pink bars) as explained in **Fig. 2A**. Mice were either sacrificed after four weeks of CPZ treatment (indicated by solid purple boxes) to assess demyelination, or after five weeks of CPZ followed by one week of normal diet (solid green boxes) to evaluate remyelination. Absolute numbers of immune cells (CD45+) (**A**), monocytes (CD11b+Ly6C+) (**B**), B cells (CD3-CD19+) (**C**), T cells (CD3+) (**D**) (further divided into CD4+ and CD8+ T cells), NKT cells (CD3+NK1.1+) (**E**) and NK cells (CD3-NK1.1+DX5+) (**F**) in CC of *WT* and *Mmp9^-/-^* mice upon receiving CPZ compared to ND mice during demyelination and remyelination (at least n=5 mice/group of 2 independent experiments). Bars represent the mean, symbols represent individual mice, and error bars indicate the standard error of mean. P values less than 0.05 were considered significant (* p < 0.05, ** p < 0.01, ****, p < 0.0001, ns non-significant). CC, corpus callosum; CPZ, cuprizone; CSF, cerebrospinal fluid; MMP, matrix metalloprotease; ND, normal diet; NK, natural killer; *WT*, wild-type.

**Suppl. Fig. S7 Expression levels of chemokine receptors on immune cells in corpus callosum of *WT* CPZ and *Mmp9^-/-^* CPZ compared to ND mice.** CPZ-induced demyelination model was induced in *WT* (grey bars) and *Mmp9^-/-^* mice (pink bars) as explained in **Fig. 2A**. Mice were either sacrificed after four weeks of CPZ treatment (indicated by solid purple boxes) to assess demyelination, or after five weeks of CPZ followed by one week of normal diet (solid green boxes) to evaluate remyelination. MFI of CCR2 of IBA1+ cells (**A**), MFI of CCR6 of B cells (**B**), MFI of CCR2, CXCR3 and CCR6 of T cells (**C**), MFI of CCR6 of NKT cells (**D**) and MFI of CXCR3 of NK cells (**E**) of *WT* and *Mmp9^-/-^* CPZ mice compared to ND mice during demyelination and remyelination (at least n=4 mice/group of 2 independent experiments). Bars represent the mean, symbols represent individual mice, and error bars indicate the standard error of mean. P values less than 0.05 were considered significant (* p < 0.05, ** p < 0.01, **** p < 0.0001, ns non-significant). CC, corpus callosum; CPZ, cuprizone; CSF, cerebrospinal fluid; MFI, mean fluorescent intensity; MMP, matrix metalloprotease; ND, normal diet; NK, natural killer; *WT*, wild-type.

**Tables**

**Suppl. Table S1. Murine flow cytometry antibodies**

| **Antigen** | **Clone** | **Fluorochrome** | **Cat #** | **Company** |
| --- | --- | --- | --- | --- |
| **CCR2** | 5A203G11 | **BV650** | Cat # 150613 | Biolegend |
| **CCR6** | 29-2L17 | **PE Dazzle 594** | Cat # 129822 | Biolegend |
| **CD11b** | M1/70 | **APC** | Cat # 17-0112-81 | Invitrogen |
| **CD19** | 6D5 | **PE Cy5** | Cat # 115510 | Biolegend |
| **CD3** | 17A2 | **PE** | Cat # 100206 | Biolegend |
| **CD3** | 17A2 | **PE Cy5** | Cat # 100274 | Biolegend |
| **CD4** | RM4-5 | **BUV737** | Cat # 612844 | BD Biosciences |
| **CD45** | 30-F11 | **BUV395** | Cat # 564279 | BD Biosciences |
| **CD49b** | DX5 | **APC** | Cat # 17-5917-82 | Invitrogen |
| **CD8** | 53-6.7 | **PerCp Cy5.5** | Cat #100734 | Biolegend |
| **CXCR3** | CXCR3-173 | **BV421** | Cat # 126529 | Biolegend |
| **F4/80** | T45-2342 | **BV711** | Cat # 565612 | BD Biosciences |
| **IBA1** | - | **Coralite 594** | Cat # CL594-10904 | ThermoFisher Scientific |
| **KI-67** | 11F6 | **PE** | Cat # 151210 | Biolegend |
| **Ly6C** | HK1.4 | **BV711** | Cat # 128037 | Biolegend |
| **Ly6C** | HK1.4 | **PerCp Cy5.5** | Cat # 128012 | Biolegend |
| **NK1.1** | PK136 | **AF488** | Cat #108718 | Biolegend |
| **NK1.1** | PK136 | **PE Cy5** | Cat # 108716 | Biolegend |
| **SPP1** | - | **Coralite 488** | Cat # CL488-22952 | ThermoFisher Scientific |

**Suppl. Table S2. RT-qPCR primers**

| **Antigen** | **Assay ID** | **Company** |
| --- | --- | --- |
| *Ccl4* | Mm.PT.58.5219433 | IDT |
| *Ccl5* | Mm.PT.58.43548565 | IDT |
| *Cntf* | Mm.PT.58.32700675 | IDT |
| *Ifng* | Mm.PT.58.41769240 | IDT |
| *Il1b* | Mm.PT.58.41616450 | IDT |
| *Il6* | Mm.PT.58.10005566 | IDT |
| *Mbp* | Mm.PT.58.28532164 | IDT |
| *Mmp2* | Mm.PT.58.7728513 | IDT |
| *Mmp12* | N008605.1.PT.Mmp12 | IDT |
| *Plp* | Mm.PT.58.28833923 | IDT |
| *Tgfb* | Mm.PT.58.8169936 | IDT |
| *Tnf* | Mm.PT.58.12575861 | IDT |
